# Severe Necrotizing Adenovirus Tubulointerstitial Nephritis in a Kidney Transplant Recipient

**DOI:** 10.1155/2013/969186

**Published:** 2013-08-28

**Authors:** Ravi Parasuraman, Ping L. Zhang, Dilip Samarapungavan, Leslie Rocher, Alan Koffron

**Affiliations:** ^1^Division of Transplantation and Department of Pathology, Royal Oak, MI 48703, USA; ^2^Oakland University William Beaumont School of Medicine, William Beaumont Hospital, Royal Oak, MI 48703, USA; ^3^Beaumont Health System, Royal Oak, MI 48703, USA

## Abstract

Adenoviruses (AdV) are emerging pathogens with a prevalence of 11% viruria and 6.5% viremia in kidney transplant recipients. Although AdV infection is common, interstitial nephritis (ADVIN) is rare with only 13 biopsy proven cases reported in the literature. We report a case of severe ADVIN with characteristic histological features that includes severe necrotizing granulomatous lesion with widespread tubular basement membrane rupture and hyperchromatic smudgy intranuclear inclusions in the tubular epithelial cells. The patient was asymptomatic at presentation, and the high AdV viral load (quantitative PCR>2,000,000 copies/mL in the urine and 646,642 copies/mL in the serum) confirmed the diagnosis. The patient showed excellent response to a combination of immunosuppression reduction, intravenous cidofovir, and immunoglobulin therapy resulting in complete resolution of infection and recovery of allograft function. Awareness of characteristic biopsy findings may help to clinch the diagnosis early which is essential since the disseminated infection is associated with high mortality of 18% in kidney transplant recipients. Cidofovir is considered the agent of choice for AdV infection in immunocompromised despite lack of randomized trials, and the addition of intravenous immunoglobulin may aid in resolution of infection while help prevention of rejection.

## 1. Introduction

Adenoviruses (AdV) are emerging pathogens in solid organ transplant recipients with clinical manifestation that ranges from subclinical infection to fatal outcome. The reported prevalence of AdV infection during the first year after kidney transplant (KT) is 11% by urine culture and 6.5% by serum PCR [1, 2]. Manifestations of urinary tract involvement may include hemorrhagic cystitis, ureteral obstruction with hydronephrosis, acute tubular necrosis, interstitial nephritis, or a mass lesion in the kidney [3–5]. Adenovirus interstitial nephritis (ADVIN) is rare in kidney transplant recipients with 13 biopsy proven cases reported in the literature [6–8]. We report a case of severe necrotizing ADVIN with characteristic morphology on biopsy within three weeks after kidney transplantation.

## 2. Case Report

### 2.1. Clinical History and Laboratory Data

A 44-year-old African American male with end-stage renal disease from hypertensive nephrosclerosis received a four-antigen mismatch, flow crossmatch negative deceased donor kidney transplantation. The patient received IL-2 receptor antagonist (Basiliximab) for induction and tacrolimus, mycophenolate mofetil (MMF), and prednisone for maintenance immunosuppression. The serological status for cytomegalovirus (CMV) was donor positive/recipient negative, and the patient received trimethoprim-sulfamethoxazole and valganciclovir for infection prophylaxis. After the transplant, the patient developed slow graft function (definition: serum creatinine (SCr) >3.0 mg/dL (265.2 *μ*mol/L) on day 5 without requiring dialysis). Subsequently, allograft function improved with SCr decreasing to 2.33 mg/dL (205.97 *μ*mol/L, eGFR 38 mL/min/1.73 m^2^) on day 19. On subsequent followup, SCr increased to 2.81 mg/dL (248.40 *μ*mol/L, eGFR 30 mL/min/1.73 m^2^) on day 22 and his urinalysis showed persistent microscopic hematuria (RBC 10–100 cells/*μ*L) with few atypical epithelial cells with no definite decoy cells. Since the rise in SCr could not be attributed clinically to volume status or tacrolimus toxicity (trough levels remained between 8 and 10 ng/mL), allograft ultrasound and biopsy were performed on day 24. The ultrasound showed an unexpected increase in echogenicity with poor corticomedullary differentiation, but the perfusion and resistive indices (from 0.54 to 0.63) were normal.

### 2.2. Kidney Biopsy

**Figure 1:**
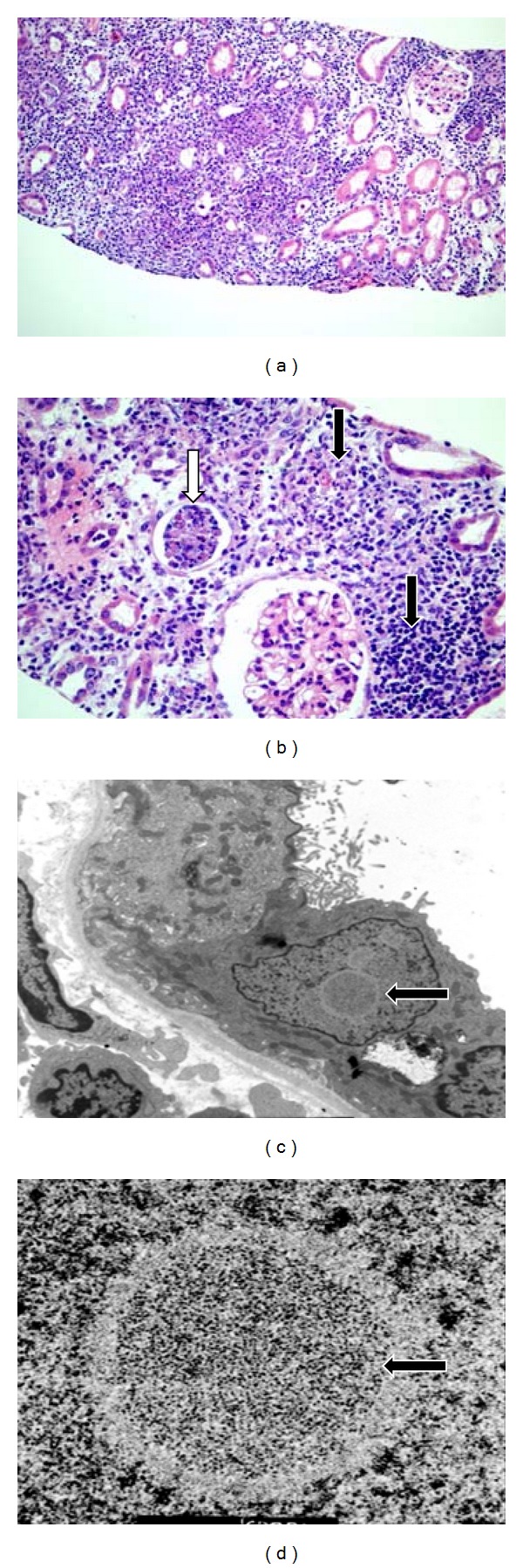
Adenovirus tubulointerstitial nephritis. Light microscopy (H&E stain): (a) Severe diffuse interstitial inflammation, (Mag ×200). (b) Granulomatous necrotizing lesions (black arrows). Inflammatory and tubular epithelial cell cast (white arrow) (Mag ×400). Electron microscopy: (c) viral inclusion body in a tubular epithelial cell (×9,500). (d) Viral particles of varying densities (×80,000).

**Figure 2:**
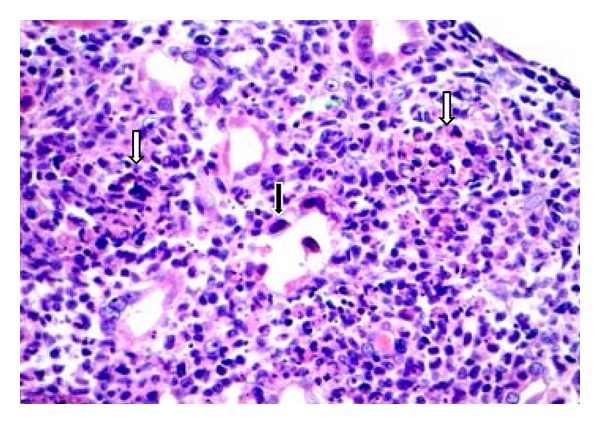
Adenovirus tubulointerstitial nephritis. Light microscopy (H&E stain): renal allograft biopsy: shows severe diffuse necrotizing granulomatous interstitial nephritis (white arrows). Basophilic, smudgy nuclear inclusions in the tubular epithelial cells (black arrow) (high power ×400).

**Figure 3:**
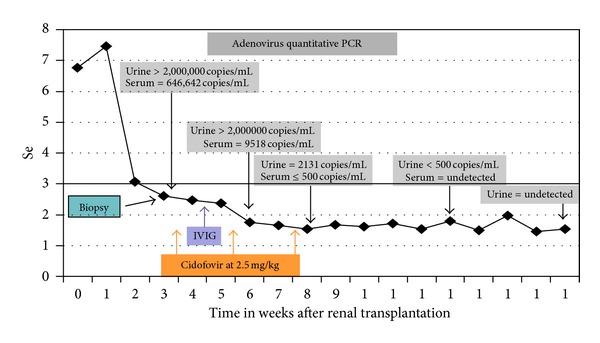
Time line and clinical course after transplantation.

Renal allograft biopsy showed a diffuse severe inflammation consisting of mostly macrophages, neutrophils, and lymphocytes with few noncaseating granulomatous lesions (Figures 1(a) and 1(b)). The most unique feature was the presence of extensive necrosis and basophilic hyperchromatic smudgy intranuclear inclusion bodies in the tubular epithelial cells (Figure 2). Additionally, there was widespread tubular basement membrane disruption. The tubulitis was minimal with no glomerular inflammation or vasculitis. The immunostain for polyoma (BK virus) and CMV were negative. The immunofluorescence (IF) for IgG, IgA, IgM, C3, C1q, fibrinogen, kappa, and lambda in the glomeruli, and C4d stain in the peritubular capillaries were negative. Periodic acid Schiff and Jones stains were negative for bacteria or fungi. Other routine investigations such as blood and urine cultures, routine viral cultures, PCR assay for Epstein-Barr, and CMV and BK viauses were all negative. Electron microscopy (EM) showed several foci of viral particles of varying densities in the nuclei of tubular epithelial cells (Figures 1(c) and 1(d)), but the crystalloid aggregates were atypical for AdV. Because necrotizing granulomatous interstitial nephritis in the presence of smudgy intranuclear viral inclusions is considered characteristic of AdV, additional investigations such as immunohistochemical (IHC) staining, in situ hybridization, and AdV quantitative PCR in the serum and urine were requested. The IHC stain and in situ hybridization for AdV were negative, but the AdV real-time quantitative PCR (QPCR) assay showed >2,000,000 copies/mL in the urine (normal <500 copies/mL, Focus Diagnostics, MICROLAB, Cypress, CA, USA) and 646,642 copies/mL in the serum as shown in Figure 3. A clinical diagnosis of ADVIN was confirmed based on high viral load in the serum and urine with characteristic morphological findings on the biopsy.

### 2.3. Diagnosis

Necrotizing adenovirus tubulo-interstitial nephritis.

### 2.4. Clinical Followup

Subsequent to confirmation of AdV interstitial nephritis, immunosuppression was minimized significantly by discontinuation of MMF, reducing prednisone to 5 mg daily with a goal tacrolimus trough level from 4 to 6 ng/mL. Despite reduction in immunosuppression, the hospital course was transiently complicated by development of symptoms of intermittent fever, macroscopic hematuria, diarrhea, and mild shortness of breath. The chest X-ray showed bibasilar patchy opacities likely representing pneumonia. Consequently, a reduced dose intravenous cidofovir at 2.5 mg/kg because of impaired allograft function and intravenous immunoglobulin (IVIG) 500 mg/kg was initiated (Figure 3) for possible early dissemination. The patient responded to therapy with resolution of all systemic symptoms within a week. The patient received two additional doses of cidofovir biweekly which he tolerated without adverse events including nephrotoxicity. Subsequently, a vigilant increase in immunosuppression guided by AdV quantitative PCR was initiated in order to prevent the potential for allograft rejection. The allograft function gradually improved to a final SCr level of 1.53 mg/dL (132.6 *μ*mol/L, eGFR 63 mL/min/1.73 m^2^) during his followup. The timeline of interventions, resolution of AdV infection, and the improvement in allograft function are shown in Figure 3.

## 3. Discussion

 Adenoviruses are nonenveloped double-stranded DNA viruses that typically cause self-limiting respiratory and gastrointestinal disease in immunocompetent individuals [9]. Recently, AdV infection is increasingly recognized in immunocompromised with high morbidity and mortality [10]. The incidence of AdV infection ranges from 3% to 47% in stem cell transplant recipients and from 5% to 22% in solid organ transplant recipients [11, 12]. AdV infection is commonly reported early after transplant when the immunosuppression is intense, and in one series 76% of all ADV infection occurred within 3 months after KT [8, 13]. AdV infection in transplant recipients may be a consequence of a primary infection, reactivation of latent infection or acquired through donor organs, and it is believed that majority of the cases are due to reactivation of latent infection [14–17]. Disseminated infection can occur in severely immunocompromised, and has been shown to be associated with poor outcome with a mortality rate of 18% in KT recipients [18]. 

Asymptomatic AdV infection is common, and the lack of symptoms during viremia has been reported in 58% of AdV infection in SOT recipients [2]. Hemorrhagic cystitis and interstitial pneumonitis are the most common clinical manifestations of AdV infection in kidney transplant recipients [19]. Renal allograft involvement is rare and can manifest as necrotizing tubulointerstitial nephritis and space-occupying lesion with or without ureteral obstruction [5, 6]. The common differential diagnoses for ADVIN include BK and CMV mediated interstitial nephritis for the most part when viral inclusions are present. However, presence of severe necrotizing granulomatous lesions with predominant neutrophilic inflammation would be considered characteristic for ADVIN [20, 21]. Additional features that are more pronounced in AdV interstitial nephritis include presence of mixed cellular infiltration, with macrophages and histiocytes, and tubular basement membrane disruption. Other differential diagnoses that should be considered for granulomatous interstitial nephritis in the absence of viral inclusions include drugs, anti-neutrophil cytoplasmic autoantibody associated vasculitis, tuberculosis, sarcoidosis, and fungal infections. Rarely, ADVIN and cellular rejection may coexist and pose a diagnostic challenge. In such situations, presence of overriding tubulitis, vasculitis, and predominant T-lymphocyte infiltration would favor presence of rejection [7]. 

Multiple diagnostic modalities may be required to clinch the diagnosis of ADVIN in cases where clinical suspicion is high. Presence of white cell casts with decoy cells on urinalysis may increase the suspicion for AdV infection. Scanning electron microscopy of urinary sediment for viral capsid and quantitative PCR for AdV DNA can confirm presence of viruria. Although tissue diagnosis is ideal for confirmation of ADVIN, not all PCR primers and antibodies used for in situ hybridization and immunohistochemistry provide complete coverage against at least 51 serotypes of adenovirus. Culture for adenovirus usually becomes positive in 2–7 days, but group D strains may take up to 4 weeks, and group F strains (serotypes 40 and 41) may not grow at all [22]. Additionally, because of the focal nature of the disease in the kidney, IHC stain and in situ hybridization may result in false negative tests. The diagnosis of ADVIN can also be confirmed by AdV QPCR assay or by typical electron microscopic finding of crystalline array particles (70–80 nm) in tubular epithelium cells when characteristic histopathological features are present in the biopsy [2, 23]. 

In contrast to the ADVIN case reported by Keddis et al., we were unable to confirm the presence of adenovirus in the biopsy specimen by IHC or in situ hybridization despite the characteristic morphology, presence of viral particles on EM, and high viral load in the urine and serum. Conversely, the EM did not show the viral particles in that case report, whereas in our case, we were able to find the viral particle on EM after extensive search [24]. These clinical scenarios of inability to demonstrate the virus at times in the pathological specimen are because of uncertainty of commercially available antibodies to capture the genetic diversity of the virus. These findings reiterate the caveats and interpretation of the results and do not essentially exclude the diagnosis when it is negative. In fact, the initial suspicion for ADVIN in our case was based on the characteristic histopathological findings.

The backbone of treatment for AdV infection in transplant recipient is reduction of immunosuppression. Although resolution of infection can occur with minimization of immunosuppression alone, cidofovir, a cytosine nucleotide analogue that inhibits DNA polymerase with greatest in vitro activity against AdV, is considered the agent of choice in immunocompromised despite the lack of randomized trials [25, 26]. However, the side effect profile particularly AKI and tubular dysfunction is a major concern that warrants consideration before its use. A less nephrotoxic new lipid conjugate of cidofovir, Chimerix (CMX001), may be of value in the future when available for commercial use [27]. 

In summary, AdV is an infrequent cause of tubulointerstitial nephritis in KT recipients and should be considered in the differential diagnosis of interstitial nephritis. Comprehension of characteristic histopathological features of ADVIN, and the caveats in its diagnosis as described in our case may facilitate an early diagnosis and better outcome. Immunosuppression reduction in all and cidofovir therapy in selected cases may significantly alter the outcome of AdV infection. Serial assessment of viral load and lymphocyte recovery are useful in monitoring the course of infection.

## Abbreviations

AdV: Adenoviruses
ADVIN: Adenovirus interstitial nephritis
QPCR: Quantitative PCR
KT: Kidney transplant
IL2-receptor: Interleukin 2 receptor
MMF: Mycophenolate mofetil
CMV: Cytomegalovirus
RBC: Red blood cells
BK virus: Polyoma
IF: Immunofluorescence
Ig: Immunoglobulins
EM: Electron microscopy
IHC: Immunohistochemical
IVIG: Intravenous immunoglobulin
SOT: Solid organ transplant
AKI: Acute kidney injury
BKVN: BK virus interstitial nephritis
ANCA: Anti-neutrophil cytoplasmic antibodies
HSCT: Hematopoietic stem cell transplant
CMX001: Chimerix.
